# Immunosenescence and Altered Vaccine Efficiency in Older Subjects: A Myth Difficult to Change

**DOI:** 10.3390/vaccines10040607

**Published:** 2022-04-13

**Authors:** Tamas Fulop, Anis Larbi, Graham Pawelec, Alan A. Cohen, Guillaume Provost, Abedelouahed Khalil, Guy Lacombe, Serafim Rodrigues, Mathieu Desroches, Katsuiku Hirokawa, Claudio Franceschi, Jacek M. Witkowski

**Affiliations:** 1 Research Center on Aging, Geriatric Division, Department of Medicine, Faculty of Medicine and Health Sciences, Université de Sherbrooke, Sherbrooke, QC J1H 5N4, Canada; abdelouahed.khalil@usherbrooke.ca (A.K.); guy.lacombe@usherbrooke.ca (G.L.); 2 Singapore Immunology Network (SIgN), Agency for Science Technology and Research (A*STAR), Immunos Building, Singapore 138648, Singapore; alarbi@beckman.com; 3 Department of Immunology, University of Tübingen, 72072 Tübingen, Germany; graham.pawelec@uni-tuebingen.de; 4 Health Sciences North Research Institute, Sudbury, ON P3E 2H2, Canada; 5 Groupe de Recherche PRIMUS, Department of Family Medicine, University of Sherbrooke, 3001 12e Ave N, Sherbrooke, QC J1H 5N4, Canada; alan.cohen@usherbrooke.ca; 6 Ikerbasque, The Basque Foundation for Science, 48009 Bilbao, Spain; guillaume.provost@usherbrooke.ca; 7 BCAM—The Basque Center for Applied Mathematics, 48009 Bilbao, Spain; 8 MathNeuro Team, Inria Sophia Antipolis Méditerranée, CEDEX, 06902 Sophia Antipolis, France; mathieu.desroches@inria.fr; 9 The Jean Alexandre Dieudonné Laboratory, Université Côte d’Azur, CEDEX 2, 06108 Nice, France; 10 Institute of Health and Life Science, Tokyo Medical and Dental University, Tokyo 113-8510, Japan; hirokawa.pth2@tmd.ac.jp; 11 IRCCS Institute of Neurological Sciences of Bologna, Alma Mater Studiorum University of Bologna, 40126 Bologna, Italy; claudio.franceschi@unibo.it; 12 Department of Applied Mathematics and Laboratory of Systems Biology of Healthy Aging, Lobachevsky State University, 603000 Nizhny Novgorod, Russia; 13 Department of Pathophysiology, Medical University of Gdansk, 80-210 Gdansk, Poland; jacek.witkowski@gumed.edu.pl

**Keywords:** immunosenescence, inflammaging, vaccination, influenza vaccine, pneumococcal vaccine, herpes–zoster vaccine, COVID-19 vaccine, immunobiography, trained immunity, adaptive complex systems, mathematical model, tipping point

## Abstract

Organismal ageing is associated with many physiological changes, including differences in the immune system of most animals. These differences are often considered to be a key cause of age-associated diseases as well as decreased vaccine responses in humans. The most often cited vaccine failure is seasonal influenza, but, while it is usually the case that the efficiency of this vaccine is lower in older than younger adults, this is not always true, and the reasons for the differential responses are manifold. Undoubtedly, changes in the innate and adaptive immune response with ageing are associated with failure to respond to the influenza vaccine, but the cause is unclear. Moreover, recent advances in vaccine formulations and adjuvants, as well as in our understanding of immune changes with ageing, have contributed to the development of vaccines, such as those against herpes zoster and SARS-CoV-2, that can protect against serious disease in older adults just as well as in younger people. In the present article, we discuss the reasons why it is a myth that vaccines inevitably protect less well in older individuals, and that vaccines represent one of the most powerful means to protect the health and ensure the quality of life of older adults.

## 1. Introduction

Vaccination is one of the greatest achievements of humankind and probably the single greatest success of modern medicine [1]. Vaccination has dramatically reduced child mortality from most of the common infectious diseases. The vaccination programme for children is extremely well organized and effective. On the other end of the spectrum of life, namely in older adults, the necessity for vaccination has become of interest for many scientists [2,3,4]. Still, there is a common thought that immunosenescence leads to a degree of immunodeficiency, which directly decreases vaccine immunogenicity as well as efficiency for all older subjects [5,6]. This opinion can be found in essentially every article and textbook treating age-related changes in the immune response and their consequences [2,3,4,5,6]. Furthermore, the alteration of the immune response is seen as responsible for not only vaccine failure in older subjects, but also for increased vulnerability to natural infections, an idea that gained even more support during the present COVID-19 pandemic due to its disproportionate impact on older subjects [7,8,9,10]. However, it should be stressed that underlying co-factors associated with ageing, such as co-morbidity, genetic and environmental factors, and overwhelming inflammaging, may play a more determinant role in COVID susceptibility than age *per se* [11].

It should be recognised that there are increasingly more vaccines proposed specifically for older subjects. However, in the beginning, the most used were the influenza and pneumococcal vaccines, which were indeed often less effective in older subjects, but were not so efficient in younger subjects either [12,13,14]. This decreased immunological efficacy was related to the changes in the immune system with ageing presently conceptualized under the concept of immunosenescence and inflammaging [15,16,17,18]. It cannot be denied by any means that immune changes occur with ageing; however, these changes cannot be treated as a monolithic block because time does not have the same effect on all humans, due in a large part to the heterogeneity of immunobiography [19,20]. This nuanced view of ageing is becoming increasingly accepted and widespread, and should direct our appreciation of vaccine efficacy in older adults [21,22].

Nevertheless, with our increased understanding of how the immune system responds to vaccines and of how the immune system changes with ageing, it has become evident that the problem was partly related to the vaccines themselves, and not to the older subjects’ immune response. No interventions can be expected to be 100% effective either in young or in older subjects. In this article, we will discuss the immune system requirements for an effective vaccine response, the immune changes related to this vaccine response during ageing, the development of new vaccines, and their usefulness in older adults.

## 2. The Immune Response Assuring an Effective Vaccine Response

Since the introduction of the first form of vaccination in the West by Jenner, there has been an enormous effort to unravel what should be the most efficient immune response for a successful vaccine response [23]. The ultimate aim of vaccination is to create a surrogate of natural infection by inducing long-lasting immune memory through coordinated and complex immunological interactions [24]. This outcome is fundamental for the protection of the organism when it again encounters the actual infectious agents. First, we briefly review the physiological immune response to vaccines before describing the changes underlying putative vaccine failure in ageing.

The antigen under any form that is injected into the organism first encounters the innate immune system or is carried directly to the lymph nodes, where the coordinated reaction of innate and adaptive immunity occurs [25,26]. The antigen-presenting cells (APCs), mainly dendritic cells (DC) and macrophages, engulf the antigen, process it into short peptides, and present it via the major histocompatibility complex (MHC) to T cells [27,28]. Adequate functioning of the innate immune system is extremely important, not only for antigen presentation, but also for the production of various cytokines, which will guide the activation of adaptive immunity and the differentiation of the different T cells [29,30].

In the adaptive arm in reaction to antigens the CD4^+^, T cell priming is the key event for vaccine immunogenicity, resulting in specific antibody production by B lymphocytes and plasma cells and the generation of long-lasting immune memory T cells [31]. This priming is highly modulated by various factors, such as the local pro-inflammatory environment, vaccine formulation, and the nature of the vaccine [32,33]. Antigens stimulate the CD4^+^ T cells depending on the cytokine milieu modulated by APC-secreted IL-12 to become either effectors or helpers for CD8^+^ cytotoxic effector T cells by the action of IL-2, IFNγ, or TNFα, or by the APC-secreted IL-10 to differentiate into Th2 and those activating B cells [34,35]. As a consequence, all of them begin to proliferate intensively [36,37,38]. The CD8^+^ T cells may also be directly stimulated by antigens in the context of MHC-I to become effector T cells. The B cells become plasma cells by the coordinated action of the follicular dendritic and CD4^+^ T cells and undergo different changes for producing highly specialized neutralizing antibodies against the antigen [39,40]. In the meantime, the clones of primed T cells that became differentiated specific effector cells will slowly shrink and ultimately die, leaving highly effective memory cells to combat future identical specific infections [36,41]. All of this complex interactive priming necessitates coordination, functionality, a large enough number of cells, functional receptors, coordinated intracellular signalling, and, finally, a solid immune memory.

This optimal immune activation chain of events that occurs the most frequently in young individuals often decreases around age 60. However, age-related immune changes occurring at any level of this coordinated action and developing through the decades were considered as detrimental and being the main reason for vaccine failure with ageing.

## 3. What Are the Changes That Are Commonly Considered to Alter the Vaccine Response with Ageing?

Ageing is not a uniform process; rather, it consists of various processes on the road of ageing [42]. This means that older subjects may age successfully with few alterations, normally with compensated changes, or pathologically with many changes in their immune functions [43,44,45,46,47]. The relatively new distinction of biological ageing from chronological ageing is also changing our understanding of ageing as it has become a time-scale-related process, where the same passage of time does not imply the same biological changes for all individuals, in accordance with the immunobiography and with the adapt-immune concept of ageing [20,48]. This is even more evident if we consider the recent appreciation of frailty as a measure of biological age [49]. Furthermore, the new approach via systems biology or the complex systems concept showed that the immune system cannot be considered cell-by-cell or cytokine-by-cytokine, but only as a whole complex, ever-adapting system [50,51,52,53]. A complex systems view is necessary to capture the unique aspects of the vaccine response of younger versus older immune systems [54]. Finally, the introduction of multi-omics approaches to capturing the multilayer components and complexity of the immune response either in populations of cells or at the single-cell level opened new ways to assess the immune response to vaccines. Very recently, our comprehension of the ageing immune response benefitted largely from these advances, either for the understanding of what is occurring in the human immune system under natural infections or under vaccine administration, as well as for the conceptualization of new vaccines [55].

What changes have been described to affect the immune response to vaccines in older subjects? Collectively, age-related immune changes are described as immunosenescence and inflammaging. Changes that could impact vaccination in the innate immune system are numerous [56,57,58,59,60,61]. The most important one is the generation of low-grade inflammation, mainly by the activation of macrophages (i.e., inflammaging) [62]. This creates an environment that is detrimental to the generation of an adequate immune response to a vaccine. This increase is partially due to the constitutive stimulation of the PRRs to produce pro-inflammatory cytokines, which renders them less effective at responding to specific stimulations [63,64,65]. Another potentially noxious event is the alteration in the antigen presentation, mainly by the DCs [66]. With ageing, these cells are unable to efficiently process and present the antigens to the T cells; additionally, the production of cytokines is not suitable for the priming of the adaptive immune response [67,68,69]. The changes observed in the lymph nodes with ageing also contribute to the altered vaccine response [25,70].

However, once the APCs are able to prime the adaptive immune response, the cells composing this arm may also be different in older individuals [71]. There are phenotypic and functional alterations. The most important phenotypic alteration is the decrease in the naïve cell numbers, mainly in the CD8^+^ T cell subpopulation, thereby precluding the priming by new antigens [72]. This is most commonly related to the thymic involution [73,74,75,76,77,78,79]. Even if the relevant cognate T cells have been found in ageing individuals, their T cell receptors (TCR) present a decrease in the signalling efficiency, either because of the membrane changes in the cholesterol content with ageing or because of the alteration in signal transduction, resulting in the less efficient transmission of the signal from the surface to the nucleus [80,81,82,83]. There are also alterations in the effector functions of the T cells, which are decreased and lead to difficulties in eliminating invading pathogens. Once the infection is resolved, memory should develop, but with ageing, instead, some effector T cells will survive, becoming either senescent or exhausted [84,85]. It seems then that, instead of becoming true memory cells, they may somehow maintain innate and effector functions, which may be somehow an adaptive process for a better immune response [84,86,87,88,89,90,91]. Most of the studies have indicated that the highly differentiated T cells, mainly CD8^+^ T cells, become senescent or even acquire senescence-associated secretory phenotype (SASP) [92,93]. However, the discussion has been ongoing for years about whether all of these cells are senescent or exhausted. Many results seem to suggest that they are also functionally exhausted, which further impairs the vaccine response [94,95]. We should nevertheless stress that the phenotypic and functional T cell subsets develop from naïve cells to memory cells through a dynamic process with underlying distinct molecular mechanisms as well as different distributions throughout the body [93,96,97]. Together, the changes observed in the cellular immune response with ageing may impact the vaccine response of the older subjects by decreasing clonal diversity due to the decrease in naïve T cells, contraction of the TCR repertoire, and the difficulties in generating long-lasting immune memory [98]. However, as per our understanding, experimental skill, and technical ingenuity are increasing, the one-way appreciation of these changes is being toned down and a more nuanced picture is appearing, favouring the building of vaccine interventions and development on the existing adaptive processes of the ageing immune system [48,99,100,101,102].

The other partner of the adaptive immune response, the B cells, is also considered to alter with ageing [103,104]. The number, the phenotype, and the functioning of the B cells change with age [105]. The switch into specific neutralizing antibodies by somatic hypermutation is changed, decreasing the ability of these antibodies to neutralize pathogens with ageing [106]. The development of efficient B memory cells is also deficient. These alterations in efficient antibody production are due to intrinsic, as well as to extrinsic (e.g., T cell), changes with ageing. All of these described changes in the adaptive immune response adversely alter the vaccine response [107,108,109,110].

The molecular underlying causes of these alterations have also been somewhat elucidated in recent years [111]. One of the most important changes is in the epigenome [112,113,114]. This closely modulates the transcription and the accessibility to chromatin. The epigenetic changes are different in CD4^+^ and CD8^+^ T cell subpopulations, which may underlie the higher susceptibility of naïve CD8^+^ T cells compared to that of naïve CD4^+^ T cells [115,116,117]. The successive differentiations induce telomere shortening contribution, but are not sufficient to induce cell senescence [88,118,119]. The overproduction of free radicals resulting from the changes in mitochondrial functions with age induces genomic instability, also leading to T cell senescence [120,121,122]. Finally, the various changes in the surface receptors induce changes in signal transduction, decreasing the efficacy of T cell activation [123,124,125,126]. Some miRNA alterations with ageing in T cells may also influence the functionality, as well as the differentiation, of T cells [127,128].

The corollary or the other side of immunosenescence is inflammaging, as first defined by C. Franceschi [17]. Because of the intrinsic and extrinsic challenges, the innate part of the immune system produces significantly increased pro-inflammatory mediators, which are not compensated by anti-inflammatory mediators [129]. This concept of macrophage-centred inflammaging has been greatly extended in recent years, with the over-activation of the adaptive immune system, senescent cells (SASP), the microbiome, and mitochondrial dysfunction being identified as contributing factors. Thus, inflammaging is suggested to be the major underlying cause of age-related chronic diseases, such as cardiovascular disease, cancer, and neurodegenerative diseases [62,100,130,131]. Furthermore, it is also well established that over-inflammation in the ageing organism decreases vaccine efficacy, either locally or systematically [132,133]. Therefore, the modulation of immunosenescence and inflammaging may be a target for increased vaccine efficacy in older adults [134].

## 4. New Evidence from Experimental Data on Vaccine Response in Old Age

One of the most important breaches in the generalized consideration of age-related immune changes as deleterious came from recent studies showing that perhaps the decrease in naïve cells due to thymic involution is not as dramatic as was assumed from murine studies. More generally, many recent studies in humans contradict longstanding concepts established from rodent research. Thus, it seems that the TCR diversity due to the low thymus activity may be compensated by the homeostatic proliferation and the stemness of some memory T cells potentially fulfilling the lifetime necessity for new TCRs during new infections [134,135,136,137,138,139]. However, very recent data indicate that the pool of naive CD8 + T cells contracts with ageing due to reduced thymic production, while the pool of naive CD4 + T cells is maintained to some extent through robust homeostatic proliferation [140]. Though this is still being debated, substantial progress has been made to better assess the clonal diversity of T cells [141]. This also agrees with the observation from clinical practice that older patients are doing much better than we could suppose considering the experimental studies. While COVID-19 is often portrayed as an example of the impacts of immune ageing, it is actually an example of the opposite: successfully ageing older adults recovered easily from this new infection, and high susceptibility appears to be more linked to comorbidities and the cumulative impacts of unhealthy lifestyles than of age itself [142]. This was confirmed by the observation that there was almost no COVID mortality in any age group in the non-industrialized Tsimane horticulturalist population, despite the high infection rates (Michael Gurven, personal communication). Of course, co-morbid frail individuals suffer serious and deadly illness from SARS-CoV-2 [143,144].

Moreover, while the number of naïve T cells may be sufficient to sustain the vaccination effects, even with a new antigen, it could be that defects in the innate immune system may hamper the effective immune response to the vaccine. However, recent experimental data supporting the notion that inflammaging may be an adaptive process in conjunction with what is called “trained innate immunity” highlights the possibility that the innate immune system could also effectively prime the adaptive immune response in older individuals [80,145,146,147]. These new discoveries suggest that better cooperation among the innate and adaptive immune response is possible in older subjects.

Recent discoveries suggest that new T cell subpopulations may exist in older subjects, namely T cells with more effector capacities, which may favour the development of better memory when the challenge is eliminated [77,88]. The new data coming from multi-omics studies concerning senescent T cells also indicate that some of them are only exhausted, which leaves the possibility to reactivate them via a blockade of checkpoint inhibitors. Furthermore, these senescent cells may retain some important effector functions, which, in turn, could be important for memory acquisition after the elimination of the pathogen [134].

What are we to make of these new findings after so many decades of research that seemed to show reduced immune functionality with age, consistent with ideas of reduced vaccine efficacy in older adults? Several concepts from complex systems theory provide plausible explanations. Most broadly, many complex biological systems show degeneracy, which is the potential to arrive at a functionally equivalent result via alternative mechanisms [148]. The best-known (but trivial) example is the degeneracy of the genetic code, with multiple codons potentially specifying the same amino acid. More relevant here, about 30% of genes, including albumin, produce no apparent change in phenotype when knocked out completely. This startling finding arises because the architecture of the underlying regulatory networks has been selected for robustness and can thus ensure the basic functioning of the system. It is likely that the ageing immune system has numerous aspects of degeneracy, which allow it to arrive at similar (emergent) functional capabilities under a wide array of immunobiographies. In fact, such degeneracy would seem absolutely necessary to maintain a functional immune system across the life course, despite the incredible heterogeneity of individual immunobiography, as reflected even in the cross-reactivity of TCR [149,150].

Degeneracy might manifest in three specific ways during immune ageing. First, there are many aspects of immune ageing that are likely adaptive. Historically, the largest risk of encountering new pathogens would mostly have been at younger ages, with some degree of saturation of memory. Counter-balanced with the risk of cancer and autoimmune disease, a reorganization of the immune system might have been actively selected for in later life. In this context, differences between young and old immune systems might be more like differences between male and female immune systems: arriving at largely similar endpoints via different pathways, with some specific differences related to the differing needs of the groups, and with some specific vulnerabilities due to the inherent trade-offs in the system [151].

Second, some immune changes with age may be pathological. Such pathological changes are likely to be diverse, depending on an individual’s immunobiography. Degeneracy may be a buffering mechanism permitting the system to persist with relatively similar overall functionality, despite deficits in certain components. Generally, in highly optimized complex systems, such buffering creates a dynamic of apparently stable systems that show a rapid or abrupt decline when their capacity is exceeded, reflecting the trade-offs needed to maintain function under the most common conditions at the expense of continual buffering capacity when the tolerance is exceeded [152,153].

Third, degeneracy could reflect the ability of the system to arrive at relatively similar functional outcomes through progressively less desirable pathways. There may be ways in which the younger immune system achieves its objectives slightly better than the older immune system, such that, as the immune system ages, it invokes numerous compensatory mechanisms for deficits that arise (either in specific individuals, or generally during ageing), but these compensatory mechanisms are partial, permitting the system to continue, but as some cost. For example, responses to certain types of pathogens might be lower, the energetic efficiency of the system might be compromised, or secondary effects, such as the consequences of cellular senescence, might be induced [154,155].

Of course, beyond degeneracy and complex systems, some aspects of the ageing immune system may also be functionally superior—most obviously, the accumulation of immunity to a greater and greater range of pathogens with age provides superior protection, even if this could not be the case for all of them. It is likely that all four of these processes (three aspects of degeneracy discussed above and the adaptive aspects of ageing) coexist, and the changes we observe in the immune system with age are a mix that we are not yet able to distinguish well. This would explain why clear decrements in many individual immune components are observed, but without a clear decrement to overall function, with major differences across individuals, and with some net generalized functional gains (e.g., increased per-cell cancer resistance) and losses (e.g., decreased influenza vaccine response) [156]. It is also consistent with continued vaccine efficacy in older adults, but with, in some cases, the need for specific formulations that work better in ageing immune systems.

## 5. How Does the ageing Immune System Respond to Various Existing Vaccines and How Do the Vaccine Modifications Improve the Response?

There are several vaccinations that are recommended for older subjects all around the world [157]. These include the influenza, pneumococcal, zoster, and tetanus vaccines, as the infections in question, as well as others that may be administered to older subjects (Table 1), are causing either serious illnesses or even being deadly in older subjects. The vaccine type recommendation, age, and mode of administration may change across countries.

The most studied vaccine is the influenza one [156,158,159]. The myth that vaccines are not efficient for the elderly population originates from the lack of success of this vaccination. Indeed, the immunogenicity and efficiency of the standard-dose influenza vaccine are about 20–50% in older adults vs. 60–90% in younger adults, depending on the season and the population [160]. The efficiency even in young people is not 100%. The standard-dose influenza vaccine contains three or four antigens from the previous influenza season produced in chicken eggs or now in insect cell cultures. This standard vaccine is administered intramuscularly and contains 15µg of each antigen. It is known not to be able to elicit efficient memory T cell responses [78,161,162,163]. The production of specific haemagglutinin-inhibition (HI) antibodies is also decreased [164,165]. These data prompted the contention that older adults do not respond to vaccines in general. However, the type of vaccine, the route of administration, and the quantity were simply not adjusted for the ageing-modified immune systems of the elderly. As these characteristics have become known, the vaccine composition has been changed. The vaccines (Fluzone High-Dose^®^, Flublok^®^) contain high doses (45 or 60µg, i.e., three or four times the standard dose) of the hemagglutinin A (HA) antigen from each of the included strains of the virus [166], becoming tetravalent, and in some cases are conjugated with a new adjuvant, M59 (e.g., Fluad^®^) [167,168,169,170]. There has been substantial improvement in the protection of older individuals with the high-dose vaccines [171]. The adjuvanted ones have not been tested directly against the high-dose vaccines, but they are significantly more efficient than the standard-dose vaccines. The alternate route of subcutaneous injection was also tested and subsequently abandoned [172]. The measure of the efficiency of the influenza vaccine is also questionable, as only an increase in the antibody titer of more than 1:40 was considered as protective, resulting in at least a 50% protection rate. The cellular immunity, notably the functionality of the CD8^+^ T cells, has not been tested or shown a real impact on functional T cell memory [173]. Together, the new vaccines against influenza are much more effective than the first generation of vaccines by inducing a strong humoral and memory T cell response [156,174,175,176]. Therefore, either the adjuvanted inactivated trivalent vaccine, the quadrivalent cell-cultured inactivated vaccine, or the high-dose tri- and tetravalent vaccines are recommended for older subjects as efficient.

The next vaccine recommended for older subjects is the vaccine against *Streptococcus pneumoniae*. The most used is the 23-valent pneumococcal polysaccharide vaccine (PPSV23), which contains the 23 most important infectious serotypes. This vaccine is highly inefficient in the elderly, either in terms of antibody production or in terms of the protection against community-acquired pneumonia (CAP). It can have some efficacy against invasive pneumococcal disease (IPD) [177,178,179,180,181]. The new conjugated vaccine, which is now most frequently used in older subjects, is the 13-valent pneumococcal conjugate vaccine (PCV13), which contains only 13 serotypes, and it is very efficient in older adults [182]; however, it may leave a place for serotype replacement [183]. The efficiency of this conjugated vaccine is very high in the older population, as demonstrated by many studies, e.g., CAPiTA [184,185]. This vaccine is able to induce protective antibody production and memory of adaptive immune cells [186,187]. It is able to reduce the occurrence of CAP in an elderly population by 74%. This vaccine is already recommended in the USA and has replaced the PPV23 alone. In the development pipeline, the PCV20 is called to replace the PCV13 mainly in the elderly to combat the serotype replacement threat [188,189]. The clinical trials of the latter vaccine are very promising in older subjects. This will probably supplant all other anti-pneumococcal vaccines in older subjects to increase their protection against this deadly pneumonia. The vaccination of children has underperformed expectations, necessitating the maintenance of strong vaccination in older adults [190].

One of the biggest successes of vaccination in older adults and a clear demonstration that vaccines can be highly efficient in this population is the adjuvanted anti-herpes zoster vaccine. The first vaccine, the Zostavax, was an attenuated virus vaccine whose efficacy waned over time because of a decrease in T cell immunity [191,192]. However, the second-generation adjuvanted anti-herpes zoster vaccine, SHINGRIX, demonstrated excellent efficacy for both its immunogenicity and its clinical efficacy [193]. Even long-term protection has been revealed remarkable, as it already lasts for 9 years [194,195]. The vaccine is composed of two components, a real viral, but recombinant, antigen, gE, involved in viral replication, and the adjuvant, AS01B, acting on the innate immune response via TLR [196,197]. The adjuvant AS01B consists of 3-O-desacyl-4′-monophosporyl (MPL) lipid A and QS-21. The efficacy of this vaccine has clearly demonstrated that, if we know what the changes in the immune system are with ageing, we are able to design sufficiently efficient vaccines to overcome the changes. This also demonstrates that a vaccine should be complex-system-oriented, and not target only one aspect of the immune response.

The other very recent vaccination success story is the unexpected efficacy of the COVID-19 vaccine in older adults [198,199,200,201]. However, the data recently published seem to indicate that age could be an important factor to explain the decrease in SARS-CoV-2 anti-S IgG after vaccination with two doses of BNT162b2 vaccine [202,203]; however, others indicate that, even if this was less in older subjects, the level of antibodies was well above what is considered protective [204]. The most recent reports all demonstrate that older subjects are responding as efficiently to the mRNA vaccine as young subjects after the third dose [205,206]. This was perceived as unexpected; however, in light of the success of SHINGRIX, it should have been expected, as the mRNA, apart from being the instruction for making the virus antigen, also acts as an adjuvant preparing a coordinated immune response, even if SARS-CoV-2 spike antigens were neoantigens [207,208,209]. Indeed, the lipid emulsion protecting the mRNA from destruction, as well as the mRNA itself, are considered as solid adjuvants. Considering this, it seems that their use is stimulating a favourable innate immune milieu, which will be able to efficiently stimulate the adaptive immune response.

## 6. Perspective on Mathematical Modelling, Illustrating the Role of Immunobiography in Vaccine Efficiency

**Figure 1:**
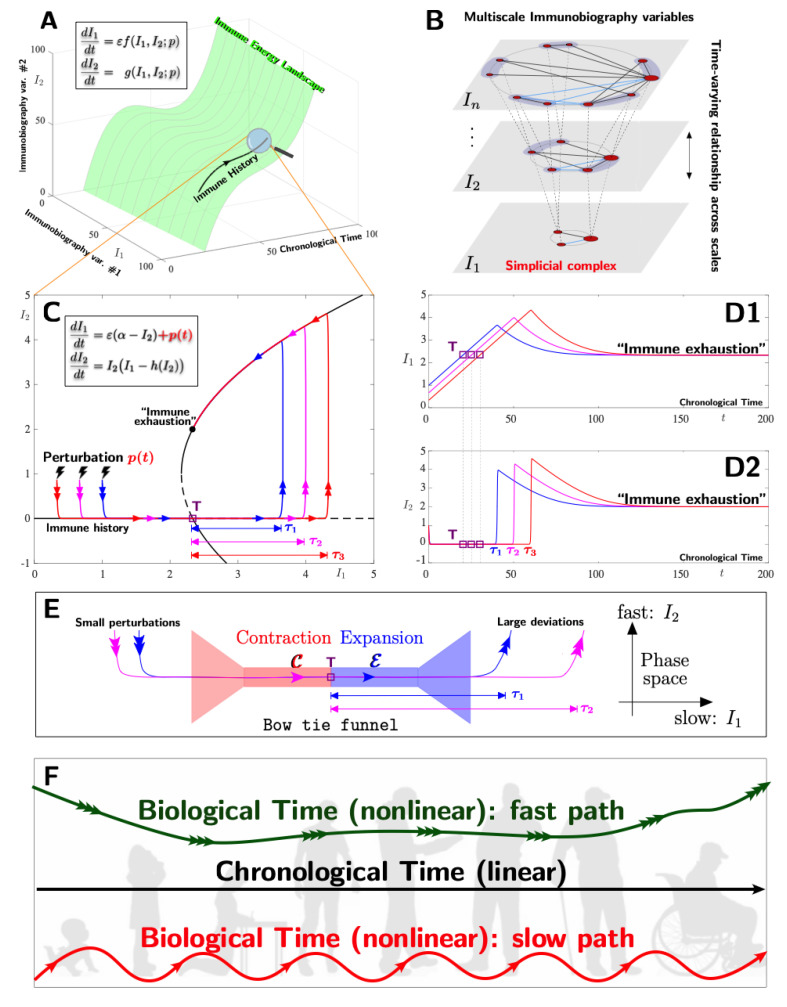
Mathematical modelling of the immune history and critical transitions towards differential ageing. (**Panel A**): Time-varying energy landscape (green) induced by the interaction between immunobiographical variables with different timescales, which is given by the differential equations; the functions *f* and *g* describe the evolution law of the immunobiographical variables, as well as their interactions. Finally, *ε* is a small parameter, a mathematical representation to capture the idea that time is contracted or dilated. The immune history evolves on this landscape (see the black trajectory segment). (**Panel B**): The immune system as an adaptive complex multiscale system, where each layer (scale) is a network or simplicial complex of interacting components. Each layer can be summarised by an order parameter *I*_i_. (**Panel C**): A specific model example of a 2-dimensional multiscale immune system; the function *h* can, for instance, be a quadratic polynomial, and *ε* and *α* are parameters. The different immune history is shown in the phase plane, where different perturbations lead to different immune history (trajectories) outcome that reach “immune exhaustion” with different time delays (i.e., τ_1_, τ_2_, and τ_3_). Note that different immune histories can be associated with different individuals or with the same individual receiving different perturbations. (**Panel D1**,**D2**): The corresponding trajectories of *I*_1_ and *I*_2_ in chronological time. (**Panel E**): A zoom of the lower part of figure C. The competition of timescales between *I*_1_ and *I*_2_ creates a funnel structure and a tipping point. Trajectories first contract onto the funnel and, initially, their biological age is not affected; however, past the tipping point T, different biological ages are induced (i.e., τ_1_, τ_2_, and τ_3_) which is dependent on small perturbations. (**Panel F**): Chronological time is linear, while biological time is nonlinear, with many components inducing either slow or fast timescales depending on the individuals and the various perturbations that they will suffer across life, indicating differential adaptations of the immune system during ageing underlying the differential vaccine response.

To provide a glimpse of what could be achieved by mathematically modelling the immune history as a complex adaptive system for demonstrating the various paths for adaptation/maladaptation that may lead to an efficient response to vaccines, we focus, for simplicity, on one feature of a complex system, namely that of multiscale properties [210]. Specifically, we consider the multiple timescale feature and assume that the immune history can be described by just two immunobiographical variables (more variables could be considered), and that these evolve on different time scales. We show that such systems are sensitive to small perturbations, and these perturbations trigger the entry towards an emergent *tipping point* that causes the differential ageing of the immune system by either precipitating or delaying the transition to “immune exhaustion” (where the immune system is less efficient in its response, but with the correct clinical intervention, the system can reactivate). To guide the reader, we substantiate these ideas with Figure 1. In Panel A, we depict two immunobiographical variables (*I*_1_ and *I*_2_, for instance, an antigen and adjuvant, respectively) that dynamically interact and generate the time-dependent energy landscape (represented by the green surface) where the immune history evolves nonlinearly in time. The laws that govern this interaction can, in principle, be described by a multi-timescale differential equation (as shown), where each of the immunobiographical variables evolves according to its own natural characteristic time. Here, *I*_1_ evolves with a slow time scale *εt* and *I*_2_ evolves with a chronological time scale *t*. The difference between the slow time scale and chronological time scale is best understood with an example: for instance, a protein–protein interaction alone may evolve at a given time scale; however, by adding an enzyme, the three components together will evolve at different time scales. Noteworthy, each of the immunobiographical variables can be seen as an order parameter, which can be thought of as a “name” that represents several components with either pairwise or higher-order (possibly time-varying) interactions (see Panel B). For example, antigens are composed of proteins, peptides, and polysaccharides. Higher-order interactions form so-called simplicial complexes [211]. These immunobiographical variables are organised in different layers *I*_1_, …, *I_n_* (i.e., a multi-layered network or simplicial complex), each characterised by a different time scale. In Panel C, we write down a specific example of a multiple-timescale differential equation (for immunobiographical variable *I*_1_ evolving with the slow time scale and *I*_2_ evolving fast), which could describe a possible scenario of the immune history within its time-dependent energy landscape. To succinctly and geometrically interpret its time history, we plot its evolution in the *phase plane*, that is, a space in which variables from different layers (of the network or simplicial complex) interact (see Panel B) and where one can identify all possible emergent states resulting from the interaction between the immunobiographical variables (in this case, *I*_1_ and *I*_2_). In this example, the interaction between *I*_1_ and *I*_2_ gives rise to two emergent states, namely, a *tipping point T* and the end state *“immune exhaustion”*. Specifically, the competition of time scales between *I*_1_ and *I*_2_ creates phase space regions geometrically akin to a bow-tie funnel structure, with both contracting and expanding directions, and, in the centre of it, there is a tipping point (see Panel E). This funnel structure attracts trajectories (i.e., acts as a magnet forcing the immune system history towards it); subsequently, the tipping point induces time delays and, finally, it expels the trajectories into different directions of the phase plane. However, the induced time delays and subsequent ejections into different directions of the phase plane are determined by the amount of initial small perturbations (e.g., pathologies, accidents, diet, lifestyle, etc.) to the immune system (see Panels C and E). In effect, small perturbations trigger the entry towards an emergent “magnet” funnel structure with a tipping point that causes the differential ageing of the immune system. The immune system inevitably reaches an end-point, “immune exhaustion”, but the uncertainty lies in the time that the immune system takes to reach “immune exhaustion”, which is determined by small perturbations and multiple-timescale interactions between the immunobiographical variables (see Panel C, where three immune history examples triggered by different perturbations lead to three different history outcomes, that is, with different delays (τ_1_, τ_2_, and τ_3_)). For simplicity, we are only considering perturbations on *I*_1_ in this example, but, in general, they could occur in every variable. The evolution of these three immune history examples is also provided in Panel D1 and D2, where we depict (in chronological time) the history of *I*_1_ and *I*_2_ respectively (Figure 1).

To summarise, the competition of timescales between immunobiographical variables leads to an immune system that is sensitive to perturbations (or initial conditions) without being chaotic and where several ageing history scenarios compete [212]. The dominant history outcome is, in fact, decided by a small perturbation. That is, different perturbations lead to differential ageing (or differential immune history), where each ageing scenario has a different delay and, consequently, reaches “immune exhaustion” at different (chronological) times. Panel F is a sketch to convey the idea that time can be contracted or dilated. This concept is summarised by showing that chronological time is linear, while biological time is nonlinear (either accelerated or slowed down by some mechanism) due to its many components with several timescales that compete and the various perturbations that an individual suffers across life, determining the immune history and age. Therefore, the efficiency of the vaccine in older subjects does not depend on the chronological age as always stated, but ultimately on the use of appropriate vaccines built on the immunobiographical adaptation related to biological ageing.

## 7. What Is the Future?

It is quite evident that, to some extent, the future is already here. By considering the changes in the ageing immune response, we are able to create efficient vaccines in older adults, as demonstrated by the anti-herpes–zoster and the anti-COVID vaccines. Therefore, more knowledge is needed to create vaccines as efficient as those against other microbes, such as HSV1, RSV, etc. We are on the right track, as many new mRNA vaccines are in the clinical trial pipeline. The development of new adjuvants is also mandatory to overcome, in some circumstances, the deleterious effect of excessive inflammaging. The reactivation of the exhausted T cells, if achievable, may be also a new avenue of improvement of vaccine efficacy, as was shown for checkpoint inhibitors in cancer treatment.

Again, the better way to design new, efficient vaccines is to better understand the ageing immune response [77,88]. The new avenues to investigate, in our comprehension, the immune changes include the role of negative regulation by Tregs and myeloid-derived suppressor cells (MDSCs) [213,214,215,216]. We should not consider it only as a deleterious process, but as a dynamic process that tries to adapt the immune response to the new circumstances of longer life, as well as towards the intensity and type of the stresses from inside and outside [217,218]. Thus, the immune response in ageing should be considered as dynamically evolving between adaptation and maladaptation. Therefore, we should use what is adaptive and overcome what is maladaptive.

The new appreciation of frailty is also fundamental to be able to reinforce the immune response to vaccines of this part for the ageing population [219]. The fact that frailty may be considered as a surrogate for biological ageing may help to design interventions when the real biomarkers of this state will be known. New composite biomarkers (e.g., immune, physiological, laboratory and epigenetic) will help to better target the alterations.

**Figure 2:**
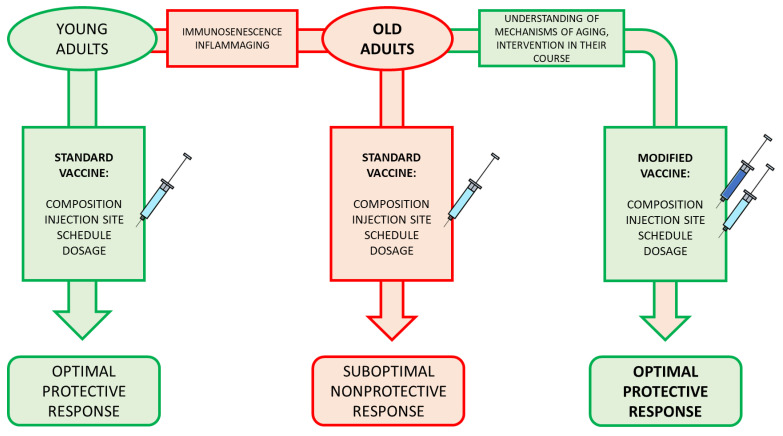
When we age from young adults to old adults, we experience immunosenescence and inflammaging, which impact our response to vaccinations, making it suboptimal (red track). However, if studies on the mechanisms of ageing (esp. immune system ageing) give us the targets (described in the text), we may intervene, on one hand, in the processes of inflammaging and immunosenescence, and, on the other, by modifying the vaccine to better suit old subjects (green track).

More importantly, immune ageing should be considered in the frame of a complex system [220]. A complex system is an open system that exchanges matter, energy, and information with its environment (and possibly stores some of these) in such a way that it does useful work to be far from thermodynamic equilibrium. It is composed of multiple components whose interaction leads to the emergence of a new behaviour that each component alone cannot generate (i.e., its behaviour is more than the sum of its parts). The interactions can be pairwise, as in standard networks, but they can also be of higher order, as in simplicial complexes, and can change over time (i.e., plastic). Complex systems may have different features, such as multi-dimensional, spatial–temporal scale, nonlinear, spontaneous order, adaptation, and feedback loops, among others [221,222]. For example, feedback loops in complex networks are distributed, rather than centralized, and provide a mechanism to stabilize or destabilize complex oscillations (or behaviours or functions). Biological systems are endowed with several of these features and, in particular, with those that they allow to self-regulate (e.g., by making internal changes) or optimize by responding to changes from their environment. That is, biological systems are complex, adaptive systems. Complex, adaptive systems have the ability to synergistically combine internal and external (environmental) information, energy, and matter in a way to optimize their performance, to evolutionarily adapt, and to survive (Figure 2). The immune system is such a system. We need a thorough study from this angle on the immune response of the older subjects. Recent studies tried to incorporate the many levels and layers from inside as well as from outside of the immune response. From these studies incorporating multi-omics approaches, AI tools, and other innovative approaches, a fuller picture will emerge, helping to better understand the immune system’s functioning and leading to the creation of new vaccines [223,224,225,226,227].

## 8. Conclusions and Perspective

Contrary to the general view of the degeneration of the immune response with ageing, new studies demonstrate that it is concomitantly adaptive and maladaptive. The outcome depends on the balance of these two entities. The new vaccine successes in older populations also reinforce that reserves still exist, which may be exploited by new vaccines. They can build concomitantly to the vaccine improvement by exploiting the mechanisms of senescence, exhaustion, and memory development, as well as trained innate immunity [228,229,230,231,232].

Future vaccines will probably build on our knowledge and will lead to immunologically and clinically efficient vaccines. Besides well-known changes in composition, adding of adjuvants, or the changes in doses, more mechanistic interventions may be implemented, such as the use of IL-7, the modulation of transcription factors and/or noncoding RNAs by the CRISPR technologies, and the use of computational models to design better vaccine targets to build on what is functioning, rather than only considering what is not.

## Figures and Tables

**Table 1:** Past and present vaccines for older subjects considering their clinical efficiency.

Vaccines	Younger Individuals	Older Individuals
Influenza		
Standard dose	+/−	-
High dose	+	++
Herpes Zoster		
Zostavax	NIL	+
Shingrix	NIL	++
SARS-CoV-2 (after 3rd dose)	+	+
Pneumococcus		
Polysaccharide	+/−	-
Conjugated	+	+
Yellow fever	+	+
Hepatitis B virus	+	+
Japanese encephalitis virus	+	+

-: almost not efficient in older individuals; +/−: efficient vaccine, but not for everybody; +: efficient in most individuals (young or elderly); ++: very efficient in almost all older subjects.

## Data Availability

Not applicable.
